# The bird’s-eye view on chromosome evolution

**DOI:** 10.1186/s13059-018-1585-z

**Published:** 2018-11-23

**Authors:** Guojie Zhang

**Affiliations:** 1 Section for Ecology and Evolution, Department of Biology, University of Copenhagen, DK-2100 Copenhagen, Denmark; 2 Center for Excellence in Animal Evolution and Genetics, Chinese Academy of Sciences, Kunming, 650223 China; 3 State Key Laboratory of Genetic Resources and Evolution, Kunming Institute of Zoology, Chinese Academy of Sciences, Kunming, 650223 China; 4 China National Genebank, BGI-Shenzhen, Shenzhen, 518083 China

## Abstract

The study of the evolution of chromosomal number and structure has long been of interest to evolutionary biologists. However, this research has been hindered by the lack of chromosome-level genome assemblies for multiple species across phylogenetic lineages. Three recent studies from same research group have demonstrated the power of bioinformatic approaches in producing chromosome level genome assemblies and reconstructing the karyotypic history of birds.

## Introduction

The evolutionary geneticist Theodosius Dobzhansky studied the chromosomal inversions in Drosophila, and proposed that chromosome evolution is a potential driver of speciation and diversification [1]. This hypothesis is corroborated by the observation that chromosomal evolution frequently accompanies speciation events as seen in cytogenetic studies in many taxa. The sequence-based characterization of chromosomal structure variation within and between species has only become possible recently with advances in genome sequencing technology. Previously, although hundreds of eukaryotic genomes have been published, a chromosome level genome assembly has only been available for a limited number of model organisms. These are of very heterogeneous ancestry and are hard to directly compare for obtaining the phylogenetic landscape of chromosome evolution. This hinders understanding of the cause and consequence of the chromosomal changes.

### Towards chromosome-level assembly

The emergence of ‘next-generation sequencing’ technologies have dramatically boosted the production of draft genome assemblies, which typically have long sequence scaffolds ranging from thousands of basepairs to several megabases. Nevertheless, only a small proportion of these genome assemblies have been anchored onto chromosomes with the assistance of genetic linkage mapping data or physical mapping data. Genetic linkage mapping infers the physical distance of genetic markers based on their recombination frequency during meiosis thus requires the collection of genome-wide genetic markers from multiple individuals of the same family, which may not be possible for species sampled in the field or with a long generation time. Physical mapping data can be obtained with fluorescent in-situ hybridization (FISH) mapping for known genetic markers, or high throughput optical mapping technologies such as BioNano. FISH, however, is less favorable because it is labor intensive and time-consuming. Optical mapping technologies can, in principle, produce one-to-one chromosome mapping for restriction sites, but its full potential crucially relies on the isolation of high molecular weight DNA which is challenging for many species for various reasons such as unavailability of fresh samples, low biomass etc. This has prevented the wide use of this technology. The sequencing-based Hi-C approach has been recently introduced to produce chromosome-level scaffolds based on the principle that the contact frequency of two genomic regions by folding strongly depends on their distance on sequence [2]. Unfortunately, this approach also produces certain amount of intra- and inter-chromosome mis-joins that result in misassembly and requires the correction with other approaches [3].

A recent study by O’Connor et al. used alternative approaches based on bioinformatics to improve the genomes of three bird species, saker falcon, budgerigar, and ostrich, into chromosome level assembly [4]. They first applied a reference assistance approach with two existing chromosome-level bird genomes, zebrafinch and chicken, as references to identify the predicted chromosome fragments (PCFs) and the chimeric scaffolds for the species of interest. The chimeric scaffolds were then either confirmed or corrected with PCR validation. And finally, the refined PCFs were anchored onto chromosome with a panel of BAC clones. The outcomes from this method have impressively improved the original genome assemblies with NGS technologies for four to eight-fold. Over 79% of the ostrich genome, 93% of the budgerigar genome and 90% of the saker falcon genome have been placed onto chromosomes. These results demonstrate the power of using comparative genomic approach to improve the genome assembly and provide a good argument that an in-silico approach offers a great addition to other approaches.

### A glimpse of avian-karyotypic evolutionary history

Bird genomes possess several unique features compared with the genomes of other vertebrate groups. They show little cross-species variation in size, ranging 0.8–1.3 Gb. The overall genome structures have been conserved at both karyotypic and microsynteny level across entire avian class for over 150 million years since their common ancestor [5]. Another recent study published by the same group found that some of these gene synteny in birds already existed in other non-avian reptile groups and had been conserved for over 225 million years [6], suggesting that such avian-like karyotype evolved before the evolution of flight that it might not be correlated with flight evolution. Nevertheless, we still cannot exclude that the stasis of such compact and ‘streamline’ genome structure might be favored by natural selection in the avian class. A previous study reported millions of avian-specific CNEs in bird genomes that have putative regulatory functions in creation of avian-specific traits, such as wing and feather development [7]. These CNEs might have formed regulatory networks that contribute to the genome structure stability in birds. O’Connor and Farre et al. (X 4) confirmed this by finding that the evolutionary breakpoint regions (EBRs) harbored a lower number of conserved non-coding element (CNEs), suggesting that the density of CNEs might define the breakpoint regions of genome structural rearrangements.

The overall stasis of the genome structure in birds raised an obvious paradox that the birds have evolved extraordinary phenotypic diversity with few genome rearrangements. In another paper reported by the same group retraced the karyotypic evolutionary history for bird class using these new chromosome-level genome assemblies [8]. The authors applied comparative genomic approaches taking phylogeny in account to construct the overall genome structure and identify lineage specific variations for 14 key nodes of birds. Despite of overall conservation across all birds, the authors reported a varied occurrence frequency of genome structural changes across bird lineages. For instance, the common ancestor of Neoaves, which includes over 95% of extant species, experienced a significantly higher rate of genome rearrangements compared with other bird groups. The authors also found a heterogeneous rate of rearrangement between macro-chromosomes and micro-chromosomes. The small chromosomes experienced higher rate of rearrangement than larger chromosomes in more recent evolution. Of particular interesting, the three most evolutionary stable avian ancestral chromosomes were all micro-chromosomes.

## Conclusion

Thus far, there is no perfect solution available to produce a chromosome level fine-map assembly. The recently initiated Vertebrate Genome Project (VGP) has set a target to a produce new reference genome assembly for 260 vertebrate orders with similar quality as the human genome, and proposed a technical roadmap to achieve this by integrating various sequencing technologies [9]. These three studies demonstrate that comparative genomic approaches could be useful complement tools to improve and accurate the assembly in additional to sequencing approaches. The application of these tools in bird genomes have revealed novel insights into the history and patterns of chromosome evolution in several bird lineages. With more extensive genome data to be generated for entire clades of species, such as the effort in B10K project which aims to produce reference genomes for all extent 10,500 bird species [10], we will be able to produce a comprehensive landscape of genome structural evolution across entire clades and ultimately address Dobzhansky’s hypothesis about the role of chromosome evolution on speciation and diversification.
